# Diagnostic accuracy of computed tomography angiography for the exclusion of coronary artery disease in candidates for transcatheter aortic valve implantation

**DOI:** 10.1038/s41598-019-56519-3

**Published:** 2019-12-27

**Authors:** Christopher Strong, António Ferreira, Rui Campante Teles, Gustavo Mendes, João Abecasis, Gonçalo Cardoso, Sara Guerreiro, Pedro Freitas, Ana Coutinho Santos, Carla Saraiva, João Brito, Luís Raposo, Pedro de Araújo Gonçalves, Henrique Mesquita Gabriel, Manuel de Sousa Almeida, Miguel Mendes

**Affiliations:** 1 Hospital de Santa Cruz, Cardiology department, Lisboa, 2790-134 Portugal; 2 Hospital de Santa Cruz, Radiology department, Lisboa, 2790-134 Portugal

**Keywords:** Interventional cardiology, Ischaemia

## Abstract

Coronary CT angiography (CTA) is currently considered a reliable method to exclude obstructive coronary artery disease (CAD) before valvular heart surgery in patients with low pretest probability. However, its role in excluding obstructive CAD before transcatheter aortic valve implantation (TAVI) is less well established. Single-center retrospective study where patients with severe symptomatic aortic stenosis underwent both CTA and invasive coronary angiography (ICA) as part of TAVI planning. CTA exams were conducted on a 64-slice dual source scanner, with a median interval of 45 days to ICA (IQR 25–75 [13–82]). In both tests, obstructive CAD was defined as a ≥50% stenosis in an epicardial vessel ≥2 mm diameter. Per-patient, per-vessel and per-proximal segment analyses were conducted, excluding and including non-evaluable segments. The study included 200 patients (120 women, mean age 83 ± 6 years). The prevalence of obstructive CAD on ICA was 35.5% (n = 71). On a per-patient analysis (assuming non-evaluable segments as stenotic), CTA showed sensitivity of 100% (95% CI, 95–100%), specificity of 42% (95% CI, 33–51%), and positive and negative predictive values of 48% (95% CI, 44–51%) and 100% (95% CI, 92–100%), respectively. CTA was able to exclude obstructive CAD in 54 patients (27%), in whom ICA could have been safely withheld. Despite the high rate of inconclusive tests, pre-procedural CTA is able to safely exclude obstructive CAD in a significant proportion of patients undergoing TAVI, possibly avoiding the need for ICA in roughly one quarter of the cases.

## Introduction

The prevalence and prognostic significance of coronary artery disease (CAD) among patients with severe aortic stenosis (AS) undergoing transcatheter aortic valve implantation (TAVI) warrant the systematic evaluation of the coronary anatomy^1–3^. Cardiac computed tomography angiography (CTA) is usually performed before TAVI for procedure planning, but may also provide valuable information about the coronary arterial tree^4^. Despite several limitations in this setting, pre-procedural CTA may be sufficient to exclude significant CAD, thus avoiding the risks and costs of invasive coronary angiography (ICA)^5–11^.

The purpose of this study was to evaluate the diagnostic performance of pre-procedural CTA for excluding significant CAD in patients with severe AS assessed for TAVI, and to determine the proportion of patients where ICA could safely be avoided.

## Results

The baseline characteristics of the 200 selected patients with severe symptomatic AS are presented in Table 1.

**Table 1:** Baseline characteristics of the patients included in the study.

	Total	Invasive coronary angiography		p value
		No obstructive CAD	Obstructive CAD	
No. of patients	200	129 (64.5%)	71 (35.5%)	
Female gender	120 (60.0%)	85 (65.9%)	35 (49.3%)	**0**.**02**
Age, years	83.4 ± 5.9	83.1 ± 5.6	83.9 ± 6.2	0.50
BMI, Kg/m^2^	26.6 ± 4.7	26.7 ± 5	26.2 ± 4.2	0.09
Arterial hypertension	185 (92.5%)	118 (91.5%)	67 (94.4%)	0.58
Dyslipidemia	147 (73.5%)	94 (72.9%)	53 (74.6%)	0.87
Diabetes mellitus	56 (28.0%)	36 (27.9%)	20 (28.2%)	1.00
Smoking history	43 (21.5%)	24 (18.6%)	19 (26.8%)	0.21
PAD	14 (7.0%)	4 (3.1%)	10 (14.1%)	<**0**.**01**
Stroke/TIA	19 (9.5%)	12 (9.3%)	7 (9.9%)	1.00
Previous pacemaker	20 (10.0%)	13 (10.1%)	7 (9.9%)	1.00
AFib	67 (33.5%)	46 (35.7%)	21 (29.6%)	0.44
Hemoglobin, g/dl	12 ± 1.7	12.2 ± 1.3	11.8 ± 2.1	0.06
COPD	33 (16.5%)	24 (18.6%)	9 (12.7%)	0.32
Cr Cl (EPI), ml/min/1.73 m^2^	55.8 ± 22.4	58.6 ± 20.5	51 ± 24.9	0.08
Euroscore II	4.0 ± 2.8	3.9 ± 3	4.2 ± 2.3	0.79
AVA, cm^2^	0.7 ± 0.2	0.7 ± 0.2	0.7 ± 0.2	0.98
MG, mmHg	50.6 ± 15.2	51.4 ± 15.6	49 ± 14.8	0.84
Reduced LVEF	48 (24.0%)	28 (21.7%)	20 (28.2%)	0.31
CAC score, median and IQR [25–75]	596 [173–1308]	442 [99.5–940.8]	1178 [499–1893]	<**0**.**01**
Inconclusive CTA	135 (67.5%)	70 (54.3%)	65 (91.5%)	<**0**.**01**

AFib - atrial fibrillation; AVA - aortic valve area; BMI - body mass index; CAC score - coronary artery calcium score; COPD - chronic obstructive pulmonary disease; Cr Cl - creatinine clearance; CTA - cardiac computed tomography angiography; IQR - interquartile range; LVEF - left ventricular ejection fraction; MG - mean gradient; PAD - peripheral artery disease; TIA - transient ischemic attack.

Overall, 71 patients (35.5%) had obstructive CAD on ICA. These patients were more often male, had a higher prevalence of peripheral artery disease, higher coronary artery calcium (CAC) score values, and a higher frequency of inconclusive CTA. The prevalence of one, two and three-vessel disease was 23.0%, 11.0% and 1.5%, respectively.

On CTA, 31 patients (15.5%) had obstructive CAD and 54 (27.0%) had no significant CAD. The remainder 115 patients (57.5%) had no clearly obstructive CAD but had at least one non-evaluable coronary segment. The main reason for classifying segments as nonevaluable was severe vessel calcification. When considering non-evaluable segments as positive for a significant stenosis in a patient-based analysis, obstructive CAD was present in 146/200 patients (73.0%). Results for the diagnostic performance of CTA for obstructive CAD are presented in Table 2. CTA correctly ruled out significant CAD in 54/200 patients (27.0%), with no false-negative results.

**Table 2:** Diagnostic accuracy of computed tomography angiography compared with invasive coronary angiography in the patient-based, vessel-based and patient-based proximal segment analyses.

	TP	FP	TN	FN	Sensitivity (95% CI)	Specificity (95% CI)	PPV (95% CI)	NPV (95% CI)
**Patient-based analysis (n** **=** **200)**								
>Including those with NE segments	69	76	55	0	100% (94.8–100%)	42% (33.4–50.9%)	47.6% (44.0–51.2%)	100% (92.2–100%)
>Excluding those with NE segments	10	5	55	0	100% (69.2–100%)	91.7% (81.6–97.2%)	66.7% (46.4–82.2%)	100% (92.2–100%)
**Vessel-based analysis (n** **=** **789)**								
>Including those with NE segments	91	294	401	3	96.8% (91–99.3%)	57.7% (53.9–61.4%)	23.6% (22.0–25.4%)	99.3% (97.8–99.8%)
>Excluding those with NE segments	17	23	401	3	85% (62.1–96.8%)	94.6% (92.0–96.5%)	42.5% (32.3–53.4%)	99.3% (98.0–99.7%)
**Patient-based proximal segment analysis (n** **=** **200)**								
>Including those with NE segments	34	90	76	0	100% (89.7–100%)	45.8% (38.0–53.7%)	27.4% (24.7–30.3%)	100% (94.2–100%)
>Excluding those with NE segments	7	8	76	0	100% (59–100%)	90.5% (82.1–95.8%)	46.7% (31.2–62.9%)	100% (94.2–100%)

FN - false negative; FP - false positive; NPV - negative predictive value; TN - true negative; TP – true positive; PPV - positive predictive value.

In vessel-based analysis, CTA correctly excluded significant CAD in 401/789 vessels (50.8%), but failed to identify obstructive CAD in 3/789 vessels (0.3%). All three cases of false-negative results involved the ostium of the right coronary artery (RCA). Only one of these three patients underwent percutaneous coronary intervention previous to TAVI due to the severity of the stenosis.

Regarding CTA’s procedural data, 18.5% of the patients were in atrial fibrillation (AFib) when CTA was performed, and the mean heart rate in the entire cohort was 72.7 ± 17.0 beats per minute. The effective radiation dose was 993 ± 378 mGy.cm. The presence of AFib did not correlate with a higher number of nonevaluable segments (p = 0.87).

### Sensitivity analysis

Cardiac CTA was performed before ICA in 79 cases (39.5%). The median number of days between CTA and ICA was 45 (IQR 25–75 [13–82]). To assess the likelihood of potential bias from the fact that CTA readers were not necessarily blinded to ICA results, we performed a sensitivity analysis, where the 121 patients who underwent ICA before CTA were excluded. Specificity and positive predictive values were lower in this subset, but sensitivity and negative predictive values remained high (Table 3).

**Table 3:** Diagnostic accuracy of computed tomography angiography compared in the patient-based analysis, excluding patients that underwent invasive coronary angiography before computed tomography.

	TP	FP	TN	FN	Sensitivity (95% CI)	Specificity (95% CI)	PPV (95% CI)	NPV (95% CI)
**Patient-based analysis (n** **=** **79)**								
>Including those with NE segments	29	36	14	0	100% (88.1–100%)	28% (16.2–42.5%)	44.6% (40.4–48.9%)	100% (74.9–100%)

FN - false negative; FP - false positive; NPV - negative predictive value; TN - true negative; TP - true positive; PPV - positive predictive value.

Finally, regarding the clinical outcome data of the 34 patients without obstructive CAD on CTA who were excluded from the main analysis for not undergoing ICA (mean age 84.0 ± 7.0 years; 79.4% male), at one year there were 3 cases of sudden cardiac death (all in patients awaiting TAVI) and no cases of hospital admission for acute coronary syndrome.

## Discussion

CTA is nowadays accepted as a reasonable alternative method to exclude significant obstructive CAD before valve surgery in selected patients with a low or intermediate pretest probability of CAD^12,13^. Its use to rule out obstructive CAD in AS patients with a higher pretest probability of coronary atherosclerosis is not so firmly established. The age of patients with AS (mostly octogenarians), their high prevalence of advanced calcified CAD, the high prevalence of atrial fibrillation during scanning, and the relative contra-indication to administer beta-blockers and nitrates are among the reasons that make coronary CT particularly challenging in this population^14^. Our results suggest that, despite these limitations, pre-procedural CTA can also be used to opportunistically screen for obstructive CAD before TAVI, potentially avoiding the unnecessary risks and costs of ICA in roughly one quarter of patients. Despite its relatively low diagnostic yield, this information comes at no additional cost in terms of contrast and radiation exposure, since CTA has become mandatory before TAVI and no change in protocol is required.

The high number of inconclusive CTA results (67.5%) comes as no surprise considering the advanced age, high prevalence of cardiovascular risk factors and high CAC score in this study population. The fact that CTA acquisition was not routinely performed under nitrates or beta-blockers could also have increased the number of inconclusive results.

In a patient-based, vessel-based and proximal segment patient-based analysis, sensitivity and, particularly NPV, remained high, even when including those patients with non-evaluable segments. These results were also not significantly affected by the fact that CTA readers were not blinded to ICA results.

To our knowledge, seven previous studies have addressed the diagnostic performance of CTA for CAD before TAVI, with study populations ranging from 60 to 475 patients^5–11^. Overall, despite different methodologies, NPV values remained roughly over 90% throughout these studies. The reported prevalence of significant CAD in patients undergoing TAVI ranges between 40–75%^15^, and similar results are stated in the studies described above. Our patients displayed a slightly lower prevalence of obstructive CAD on ICA (35.5%), possibly due to the fact that known CAD was an exclusion criterion. Our study adds to the current body of evidence showing that CTA is useful for excluding obstructive CAD before TAVI.

As for safety concerns, there were three cases of false negative results on a per-vessel analysis (0.4%), all of them related to RCA ostial lesions which were considered non-significant on CTA. The time interval between CTA and ICA was small (<10 days for all three lesions), ruling out the possibility that the coronary stenosis could have progressed between the two tests. Reassessment of these three lesions in CTA suggested calcification of the aortic root wall as the cause for the misjudgement. False negative results were also not exclusive to our study, with previous studies reporting up to 1.5% false negative results in vessel-based analysis^8,10,11^.

Our study has several limitations. First, this was a retrospective study, conducted in a single center, with a limited sample size, and as such, its results may not be applicable to other populations and may not represent the exact accuracy of CTA in TAVI patients. Second, interventional cardiologists and imaging cardiologists were sometimes aware, beforehand, of the results of the ICA or CTA. However, significant bias seems unlikely since our sensitivity analysis displayed similar results to the global analysis. Third, a follow-up for the occurrence of vascular complications and contrast nephropathy resulting from ICA was not performed, and so we can only speculate on the potential harm of conducting a potentially unnecessary ICA. Fourth, the 6-month time interval between CTA and ICA defined as cut-off is wider than that stated in previous studies. However, this did not seem to affect CTA’s diagnostic performance.

In our population of elderly patients with severe symptomatic aortic stenosis, coronary CT angiography was able to safely exclude obstructive coronary artery disease in roughly one quarter of the cases. The clinical implications of these findings, taken together with similar studies, may include changes in institutional protocols for transcatheter aortic valve implantation planning. Heart teams involved in patient selection and procedure planning should consider performing coronary CT angiography as a first-line test and withholding invasive coronary angiography whenever the absence of significant stenoses can be demonstrated non-invasively. Although beyond the scope of our study, it can be speculated that such an approach might avoid the risks and costs of some unnecessary invasive angiographies without compromising patient safety.

## Methods

### Patient selection

**Figure 1:**
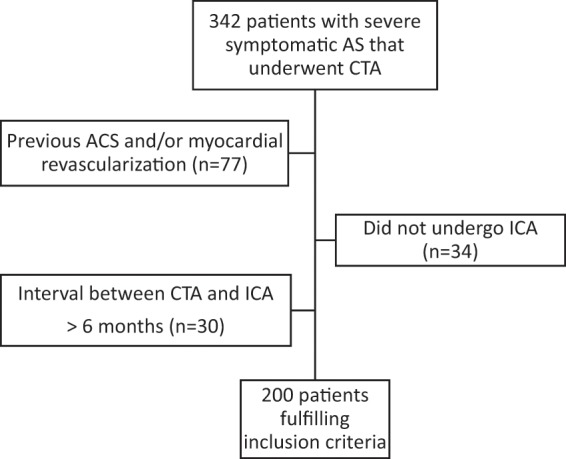
Patient selection flowchart. ACS - acute coronary syndrome; AS - aortic stenosis; CAD - coronary artery disease; CTA - computed tomography angiography; ICA - invasive coronary angiography.

In this single-center longitudinal retrospective study, all potential candidates for TAVI undergoing both pre-procedural CTA and ICA between October 2015 and March 2018 were included. Patients were considered potential candidates for TAVI based on the presence of severe symptomatic AS, estimated life expectancy greater than one year, and intermediate or high surgical risk. The TAVI screening protocol included both CTA and ICA^16^. Candidates with a time interval between CTA and ICA greater than six months were excluded. Patients with previous history of acute coronary syndrome and/or previous coronary revascularization (percutaneous or surgical) were also excluded. The patient selection flowchart did not exclude patients with poor image quality (Fig. 1). Informed consent was obtained from all subjects. The study was given approval by the *Comissão de Ética para a Saúde* of *Centro Hospitalar Lisboa Ocidental*.

### Scan protocol and image analysis

Cardiac CTA was used to assess the aortic annulus size and shape, the aortic valve anatomy and degree of calcification, the distance between the annulus and coronary ostia, the aortic and iliofemoral dimensions, and the coronary anatomy. Coronary CTA was performed on a dual source 64-slice CT (*Siemens Somatom Definition®*, *Siemens Healthineers*, Erlangen, Germany) according to the Society of Cardiovascular Computed Tomography guidelines^14^.

Briefly, a bolus of 80–100 mL of iodinated contrast was injected at 4.5 mL/s in an antecubital vein, followed by a 30–50 mL saline flush. A bolus-tracking technique was employed, with a region of interest placed into the ascending aorta, set to detect a predefined threshold of 120 HU. For cardiac imaging, an ECG-gated retrospective scan with tube current modulation was used, with full tube current applied at 25% to 70% of the R-R interval. Tube potential was set at 100 kV by default, and increased to 120 kV in patients with body mass index greater than 30 Kg/m^2^. Cardiac imaging was immediately followed by a non-gated scan of the entire aorta and femoral arteries. For the cardiac dataset, transaxial images were reconstructed with a slice thickness of 0.6 mm in at least two points of the cardiac cycle (systole and diastole). The vascular dataset was reconstructed with a slice thickness of 1.0 mm.

Coronary artery calcium (CAC) was determined using the *Agatston* score. Given the presence of severe symptomatic AS, no routine beta-blockers or nitrates were given before CTA, regardless of baseline heart rate or blood pressure. All CTA scans were analyzed by a cardiologist or radiologist with level III experience, on an *Aquarius®* workstation (*Terarecon® Inc*., USA), using axial images, multiplanar reconstructions and maximum intensity projections, as appropriate. ICA exams were performed using standard techniques and projection planes, and analysed by experienced interventional cardiologists. Coronary atherosclerotic lesions on CTA and ICA were assessed by visual estimation. Obstructive CAD was defined as the presence of any plaque with ≤50% diameter stenosis in an epicardial vessel with ≥2 mm diameter. This threshold was chosen since patients without ≥50% stenosis usually warrant no further testing^17^. The same definition was used for ICA. The diagnostic performance of coronary CTA was assessed using ICA results as gold standard on a per-patient, per-vessel and per-proximal segment basis (where only the left main and proximal segments of the three epicardial coronary arteries were considered for analysis). On the per-vessel analysis, if more than one obstructive stenosis was present in the same vessel, the most severe lesion was considered as representative of the vessel. Segments located distally to a chronic total occlusion were also excluded from the analysis. In order to deal with the presence of non-evaluable segments (segments where the scan reader could not reliably evaluate the degree of stenosis), we conducted two separate analyses: first, assuming that all non-evaluable segments had obstructive CAD, and second, excluding all non-evaluable segments from the analysis. In the vessel-based analysis of the right coronary artery, non-dominant vessels were excluded. Finally, since ICA was sometimes performed before coronary CTA and scan readers were not sistematically blinded to ICA results, we performed a sensitivity analysis excluding these patients in order to assess the likelihood of significant bias.

All methods were carried out in accordance with relevant guidelines and regulations.

### Statistical analysis

Continuous variables are presented as means and standard deviations for data with normal distribution, and as median and interquartile range for non-normally distributed data. Normal distribution of continuous data was assessed with the Kolmogorov-Smirnov test. Categorical variables are expressed as frequencies and percentages. Fisher’s exact test and unpaired t test were used to compare categorical and normally distributed continuous data, respectively. The diagnostic performance of CTA is expressed as sensitivity, specificity, positive predictive value and negative predictive values, with corresponding 95% confidence intervals, using ICA results as the reference standard. All analyses were performed using IBM SPSS software version 23. Two-tailed *p* values < 0.05 were considered statistically significant.
